# Nanocellulose Film Properties Tunable by Controlling Degree of Fibrillation of TEMPO-Oxidized Cellulose

**DOI:** 10.3389/fchem.2020.00037

**Published:** 2020-02-13

**Authors:** Moe Wakabayashi, Shuji Fujisawa, Tsuguyuki Saito, Akira Isogai

**Affiliations:** Department of Biomaterial Sciences, The University of Tokyo, Tokyo, Japan

**Keywords:** TEMPO, cellulose nanonetwork, degree of fibrillation, film properties, filtration behavior, oxygen permeability

## Abstract

A fibrous 2,2,6,6-tetramethylpiperidine-1-oxyl (TEMPO)-oxidized wood cellulose/water slurry was disintegrated with a magnetic stirrer or high-pressure homogenizer under various conditions to prepare TEMPO-oxidized cellulose (TOC)/water dispersions with different degrees of fibrillation. The turbidity value of the as-prepared dispersion was used as a measure of the degree of nanofibrillation of the fibrous TOC slurry in water. The fibrillated TOC/water dispersions with low degrees of fibrillation had cellulose nanonetwork (CNNeW) structures consisting of TOC nanofibrils (TOCNs), unfibrillated TOC fibers, and fibril bundles. The original TOC/water slurry and partly fibrillated TOC/water dispersions with low degrees of fibrillation were converted to a sheet and films, respectively, in a short time by membrane filtration, and they had low bulk densities and high porosities. Membrane filtration of an almost completely nanofibrillated TOC/water or TOCN dispersion took a long time, but the as-prepared TOCN films had the highest light transparency, tensile strength, Young's modulus, and work of fracture. The oxygen permeabilities of the films at 23°C and 50% relative humidity were as low as 1–2 ml μm m^−2^ day^−1^ kPa^−1^ among the films prepared from the fibrillated TOC/water dispersions with a wide turbidity range of 0.01–0.45. Therefore, TEMPO-oxidized CNNeW films with the versatile optical, porous, and mechanical properties but similarly low oxygen permeabilities can be prepared by controlling the degree of fibrillation of the TOC/water slurry (Graphical Abstract).

**Graphical Abstract:**
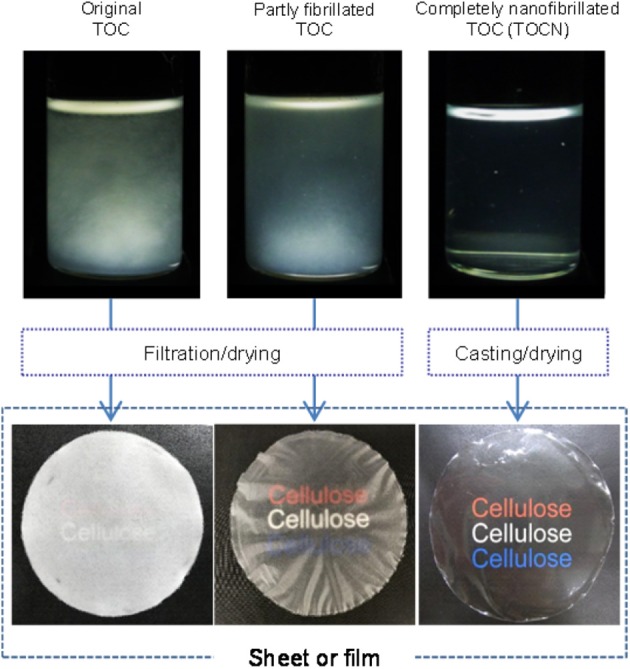
TEMPO-oxidized cellulose fiber sheet and nanofibril films.

## Introduction

The 2,2,6,6-tetramethylpiperidine-1-oxyl (TEMPO) radical is a water-soluble, commercially available, and stable nitroxyl radical. The primary C6–OH groups of polysaccharides are selectively oxidized to sodium carboxylate groups by catalytic oxidation with TEMPO in water (de Nooy et al., 1995, 1996). TEMPO-mediated oxidation of plant, bacterial, and tunicate celluloses in water at pH 7 or 10 at room temperature densely and position-selectively forms sodium C6–carboxylate groups on crystalline cellulose microfibril surfaces (de Nooy et al., 1995; Okita et al., 2009; Hirota et al., 2010; Isogai et al., 2011, 2018). Wood cellulose nanofibrils and nanocrystals with high and low average aspect ratios of >150 and <60, respectively, and homogeneous and ultrafine widths of ~3 nm can be produced from fibrous TEMPO-oxidized wood celluloses (TOCs) containing >0.8 mmol/g sodium carboxylate groups by mechanical disintegration in water under suitable conditions (Isogai et al., 2011, 2018; Zhou et al., 2018, 2019). When TEMPO-oxidized cellulose nanofibril (TOCN) films are prepared from completely individualized and transparent TOCN/water dispersions by casting on a plate or filtration on a membrane filter and subsequent drying, the films have good flexibilities, high light transparencies, and high mechanical strengths (Zhao et al., 2018), extremely low oxygen permeabilities of <0.2 ml μm m^−2^ day^−1^ kPa^−1^ under dry conditions, and low thermal expansion coefficients of <10 ppm/K (Fukuzumi et al., 2009, 2013; Zhao et al., 2018).

Because the TOCN concentrations in dispersions are lower than ~1% owing to their high aspect ratios, removal of water from TOCN/water dispersions to prepare TOCN films requires long casting/drying times or high energy consumption during filtration or thermal drying. The homogeneous TOCN/water dispersions transform to heterogeneous gels by excess evaporation of the dispersions to increase the solid concentrations, and they cannot be used for film preparation. When TOCN/water dispersions are sonicated for an extended time, such as 60–120 min, in laboratory experiments, TEMPO-oxidized cellulose nanocrystals (T-CNCs) with average aspect ratios of <60 are produced and their gelation concentrations increase to 1.7%. However, the T-CNC/water dispersions still contain a large amount of water (>98%). Therefore, development of an efficient and cost-effective removal method or process to prepare TOCN films from TOCN/water dispersions is a challenging task for application of TOCNs.

When wood cellulose fibers with or without mild pretreatment are mechanically disintegrated in water, nanocelluloses consisting of cellulose nanonetwork (CNNeW) structures differing from TOCNs or T-CNCs are produced (Henriksson et al., 2007; Pääkkö et al., 2007; Wågberg et al., 2008; Klemm et al., 2011; Moon et al., 2011; Isogai and Zhou, 2019). The CNNeWs have heterogeneous fibril widths of 5–100 nm and branched or network structures of cellulose fibrils depending on the original wood cellulose fibers and mechanical disintegration conditions. Mild endo-cellulase treatment and carboxymethylation are included in the pretreatments (Nakagaito and Yano, 2005, 2008; Abe et al., 2007; Henriksson et al., 2007, 2008; Pääkkö et al., 2007). These CNNeW/water dispersions can be efficiently converted to sheets or films in a short time by dewatering with filtration, like the papermaking process. The optical, mechanical, and thermal properties of the CNNeW sheets or films and their composites with polymers or nanoclays have been investigated in detail (Svagan et al., 2007; Eichhorn et al., 2010; Sehaqui et al., 2010, 2011, 2012; Liu et al., 2011; Varanashi and Batchelor, 2013). Although the TOCN films are superior to the CNNeW sheets and films in terms of the optical, mechanical, thermal, and oxygen-barrier properties, the CNNeW sheets and films have advantages in terms of the efficient removal of water in the dispersions by filtration and the related drying process.

In this study, a fibrous TOC/water slurry was disintegrated with a magnetic stirrer or high-pressure homogenizer under various conditions to prepare partly fibrillated TOC/water or CNNeW dispersions with various degrees of fibrillation. The filtration behavior and optical, mechanical, and oxygen-barrier properties of the sheet prepared from the TOC/water slurry and the films prepared from the partly fibrillated TOC/water dispersions were investigated to determine the relationships between the degree of fibrillation of TOC and the fundamental properties of the films. Because all the films were prepared from the same TOC, they have the same amount of sodium carboxylate groups. Therefore, all the sheet and films have the ability for ion-exchange of sodium with other metal and alkylammonium ions (Shimizu et al., 2016; Isogai et al., 2018).

## Materials and Methods

### Fibrillation of TOC Dispersion Under Various Conditions

A fibrous TOC with carboxylate content of 1.3 mmol/g prepared from softwood bleached kraft pulp was provided by Nippon Paper Industries (Tokyo, Japan). The carboxylate content was determined by the conductivity titration method (Saito and Isogai, 2004; Fraschini et al., 2017). The TOC/water slurry with TOC concentration of 0.1 wt % was disintegrated under the following conditions: stirring with a magnetic stir bar at room temperature and 500 rpm for 20 days, followed by high-pressure homogenization with a water-jet-type apparatus by collision with a ceramic ball (Starburst HJP-25005X, Sugino Machine, Toyama, Japan) for one pass at 30, 50, and 150 MPa, and five passes at 150 MPa, which are denoted ST, 30P1, 50P1, 150P1, and 150P5, respectively. Tert-butanol (*t*-BuOH) and the other chemicals and solvents of laboratory grade were purchased from Fujifilm Wako Pure Chemicals (Tokyo, Japan) and used as received. Based on the mechanical fibrillation conditions of the TOC/water dispersion, the degree of fibrillation was assumed to increase in the following order: TOC < ST < 30P1 < 50P1 < 150P1 < 150P5.

### Characterization of the Fibrillated TOC Dispersions

The fibrillated TOC/water dispersions with solid concentration of 0.1 wt% were observed with or without cross-polarizers. The light transmittance values of the dispersions were measured from 300 to 900 nm wavelength with an ultraviolet–visible (UV-vis) spectrophotometer (V-670, JASCO Co., Tokyo, Japan), and their turbidity values were measured at 600 nm wavelength. The fibrillated TOC/water dispersions (50 ml) in plastic centrifuge tubes were centrifuged at 12,000×g for 15 min. The supernatant fractions were dried at 105°C for 3 h to determine the solid weights, and the nanofibrillation yields are expressed based on the solid weights in the supernatant fractions and those in the dispersions before centrifugation. *t*-BuOH was added to the fibrillated TOC/water dispersions to adjust the *t*-BuOH concentration to 30 wt% (Nemoto et al., 2015). Here, no gels formed in the mixture. The fibrillated TOC/water/*t*-BuOH dispersions were freeze-dried. The specific surface areas of the freeze-dried products were determined with a N_2_-adsorption apparatus (NOVA 4200e, Quantachrome Instruments, Florida, USA) using the Brunauer–Emmett–Teller equation. The large fibers or fibrils present in the fibrillated TOC/water dispersions were observed with an optical microscope (BX51, Olympus, Tokyo, Japan) with cross-polarizers. The fibrillated TOC/water dispersions with solid concentration of 0.1 wt% were filtered on a polyvinylidene fluoride (PVDF) membrane filter (0.1 μm pore size, Durapore, Merck, Germany) under reduced pressure using a vacuum pump (V-300, Büchi, Germany), and the filtration times required to remove most of the water from the dispersions were recorded.

### Preparation of the Sheet and Films

The TOC/water slurry and fibrillated TOC/water dispersions were converted to a wet sheet and wet films, respectively, by filtration on PVDF membranes under reduced pressure. The pore size of the membranes used for filtration of the ST, 30P1, and 50P1 dispersions was 0.65 μm, and that for filtration of the 150P1 dispersion was 0.1 μm. The wet sheet and films formed on the membrane filters were peeled off. Some weights were placed on the wet film edges to prevent shrinkage during drying, and the wet sheet and films were dried at 23°C and 50% relative humidity (RH) for more than 1 day. In the case of the 150P5 dispersion, the film was prepared from the 150P5 dispersion by pouring on a glass Petri dish and dried at 40°C in an oven for more than 3 days.

### Characterization of the Sheet and Films

All of the following analyses were performed for the sheet and films after conditioning at 23°C and 50% RH for more than 1 day. The thickness, bulk density, water content at 23°C and 50% RH, and porosity of the sheet and films were measured. The thickness of each sample was measured at 10 different points with a micrometer and it is expressed as the average value with standard deviation. The sheet or film conditioned at 23°C and 50% RH was dried at 105°C for 3 h to determine the dry weight for calculation of the water content at 23°C and 50% RH. The porosity of the sheet or film was calculated with the following equation:

$$\begin{array}{l} \text{Porosity}=100\\ \;-\frac{\text{bulk density }\times \;\left(1-\text{water content}\right)}{\text{true density}}\times 100\;(\%) \end{array}$$

where the true density of 1.6 g/cm^3^ was used for wood TOCNs (Daicho et al., 2018). The surface roughness values of the films were measured for the film/air interface side, which formed during the film preparation process, from the atomic force microscopy (AFM) images (Wu et al., 2014). The light transmittance values of the sheet and films were measured with the V-670 UV-vis spectrophotometer and normalized to 50 μm thickness according to the Lambert–Beer law. The tensile tests of the sheet and films were performed with a tensile tester (Shimadzu Ex-TES, Kyoto, Japan) using a 500 N load cell. The sample specimens were 5 mm wide and 4 cm long, and they were tested with a span length of 1 cm at a speed of 0.5 mm/min. The oxygen transmission rates (OTRs) of the films were recorded with an oxygen permeability tester (MOCON OX-TRAN 2/21, Modern Controls Inc., USA) at 23°C and 50% RH under standard conditions (Yang et al., 2015). The OTRs were converted to oxygen permeability values according to the following equation:

$$\begin{array}{l} \text{Oxygen permeability }\left(\text{ml μm kP}{\text{a}}^{-1}\;{\text{m}}^{-2}\text{ da}{\text{y}}^{-1}\right)\\ \;=\text{OTR}\times \text{film thickness}/101.3 \end{array}$$

## Results and Discussion

### Disintegration of the TOC/Water Slurry Under Various Conditions

**Figure 1:**
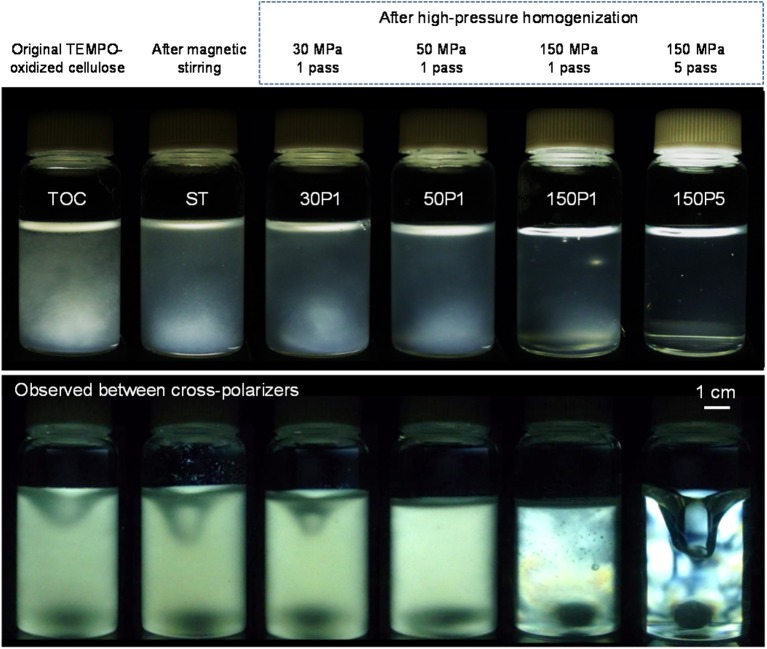
Photographs of the TOC/water slurry and fibrillated TOC/water dispersions prepared under various disintegration conditions taken with and without cross-polarizers.

The TOC/water slurry was disintegrated under various conditions, and photographs of the slurry and dispersions taken with and without cross-polarizers are shown in Figure 1. The disintegration conditions to prepare the 150P5 dispersion were representative of that of a TOCN dispersion consisting of completely individualized nanofibrils with homogeneous widths of ~3 nm. This 150P5 dispersion was transparent and exhibited typical birefringence behavior when observed between cross-polarizers owing to the individualized TOCN elements (De Souza Lima and Borsali, 2004). The other dispersions were opaque or translucent and did not show clear birefringence because of incomplete fibrillation of the fibrous TOC. Fibrous morphologies were observed in the original TOC/water slurry, and they formed a sediment mat at the bottom soon after being left to stand.

**Figure 2:**
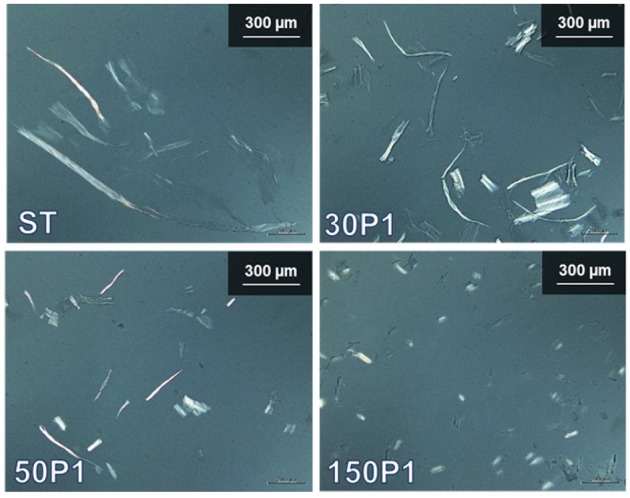
Optical microphotographs of the fibrillated TOC/water dispersions prepared under various disintegration conditions, taken with cross-polarizers.

Optical microphotographs of the ST, 30P1, 50P1, and 150P1 dispersions taken with cross-polarizers are shown in Figure 2. Because the cellulose fibers were crystalline, they were observed as bright fibers differing from the background water phase containing fine nanofibrils. The fiber length became shorter as the degree of fibrillation of TOC increased from ST to 150P1. Therefore, nanofibrillation of the TOC fibers proceeded with vertical cutting to the fiber length (Saito and Isogai, 2004).

**Figure 3:**
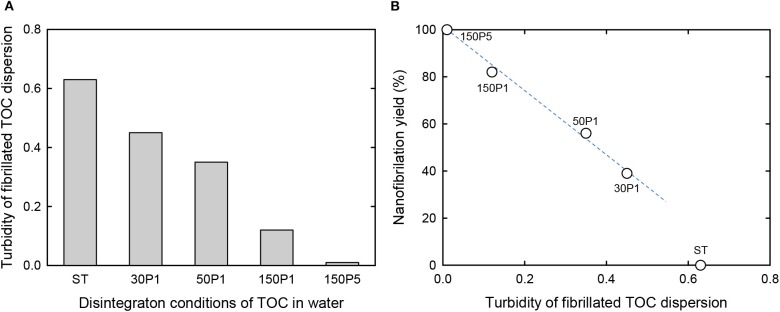
**(A)** Turbidity of the fibrillated TOC/water dispersions prepared under various disintegration conditions. **(B)** Relationship between the turbidity of the TOC/water dispersion and the corresponding nanofibrillation yield.

The turbidity values of the fibrillated TOC/water dispersions and their nanofibrillation yields are shown in Figures 3A,B, respectively. As expected, the turbidity decreased with increasing degree of fibrillation from ST to 150P5. Correspondingly, the nanofibrillation yield linearly increased from 39 to 100% with increasing degree of fibrillation from 30P1 to 150P5. Therefore, the turbidity of the fibrillated TOC/water dispersions prepared under various disintegration conditions can be regarded as a measure of the degree of nanofibrillation, although the nanofibrillation yield of the ST dispersion was outside the linear relationship.

**Figure 4:**
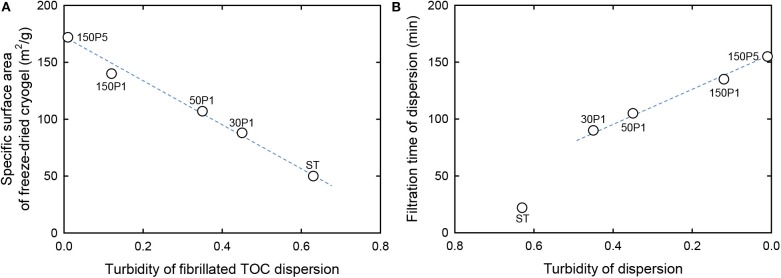
Relationship between the turbidity of the fibrillated TOC dispersion **(A)** and the specific surface area **(B)** of freeze-dried cryogels prepared from the dispersions containing *t*-BuOH.

The relationship between the turbidity of the fibrillated TOC/water dispersion and the specific surface area of the freeze-dried cryogel prepared from the fibrillated TOC/water/*t*-BuOH dispersions is shown in Figure 4. When TOCN/water dispersions are directly freeze-dried, the obtained cryogels have honeycomb structures because of growth of ice crystals during freezing (Sakai et al., 2016). In contrast, nanofibrous cryogels can be prepared from TOCN/water/*t*-BuOH dispersions containing ~30 wt% *t*-BuOH (Nemoto et al., 2015). There was an almost linear relationship between the turbidity and the specific surface area for all of the fibrillated TOC/water dispersions, including the ST dispersion.

### Preparation and Characterization of the Sheet and Films

**Figure 5:**
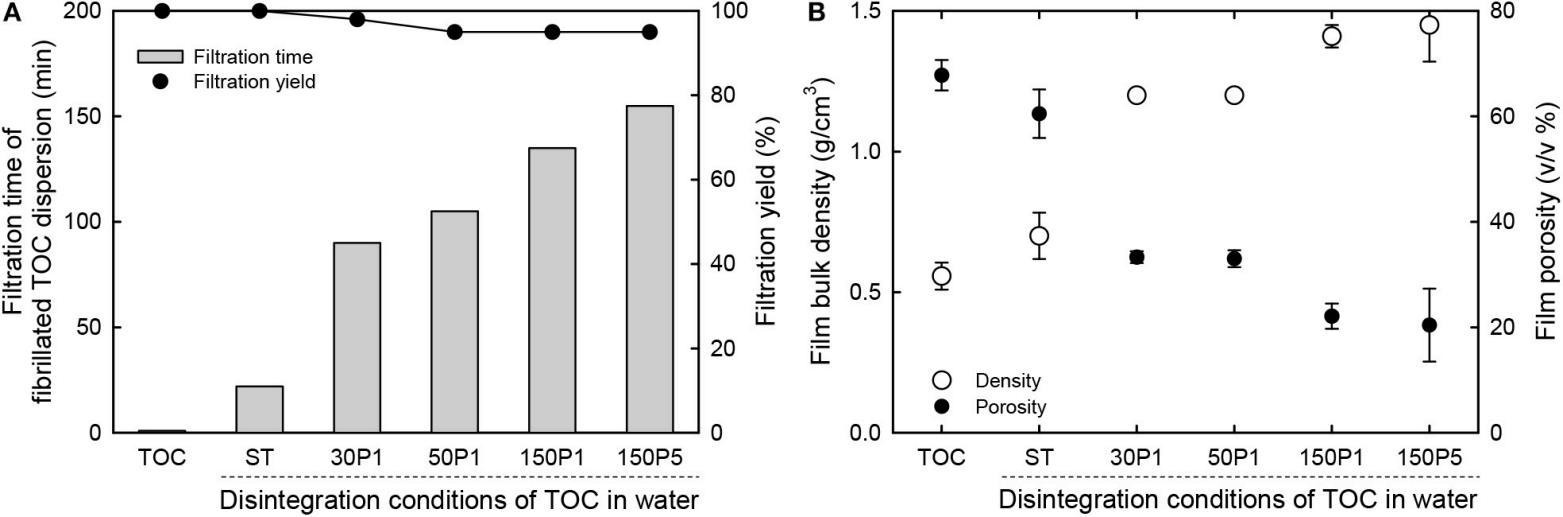
Relationships between the disintegration conditions of the TOC/water slurry and the **(A)** filtration time of the dispersion and solid component yield, and **(B)** bulk density and porosity of the film.

The original TOC/water slurry and fibrillated TOC/water dispersions were filtered on PVDF membranes. The filtration times required for removal of most of the water from the slurry and dispersions are shown in Figure 5A. As expected, the original TOC/water slurry was converted to a TOC sheet within 1 min by filtration. The ST dispersion was converted to the corresponding film within 0.4 h. The filtration time increased with increasing degree of fibrillation of the TOC/water slurry, and the 30P1, 50P1, 150P1, and 150P5 dispersions required 1.5, 1.8, 2.3, and 2.6 h filtration, respectively. Moreover, for the 50P1, 150P1, and 150P5 dispersions, the filtration yields decreased to 95%, probably because of partial passing of fine nanofibrils through the membrane during filtration.

The bulk density and porosity values of the sheet and films are shown in Figure 5B. The film density increased from 0.56 to 1.45 g/cm^3^ and the film porosity decreased from 70 to 20% with increasing degree of fibrillation of the TOC/water slurry. Hence, the film density and porosity can be controlled by controlling the degree of fibrillation. The dried TOC sheet and CNNeW films with high porosities can be used as base materials to prepare polymer composites by impregnating polymer/solvent solutions or thermally melted or softened polymers. The thickness, moisture content, bulk density, and porosity values with standard deviations of the sheet and films are given in Table 1. The moisture content increased from 7.4% for the TOC sheet to 12% for the 150P5 film, even though all the sheet and films had the same hydrophilic sodium carboxylate content of 1.3 mmol/g. Therefore, the moisture content of the film prepared from the fibrillated TOC/water dispersion increased with increasing degree of fibrillation of the TOC/water slurry. Some of the sodium carboxylate groups inside each TOC fiber in the TOC sheet did not behave as hydrophilic sites.

**Table 1:** Fundamental properties of the TOC sheet and films prepared from fibrillated TOC/water dispersions.

	**Disintegration condition**	**Thickness (μm)**	**Moisture content (%)**	**Bulk density (g/cm^**3**^)**	**Porosity (%)**
TOC sheet	–	86.6 ± 9.5	7.4 ± 0.4	0.56 ± 0.05	67.8 ± 2.9
Films prepared from fibrillated TOC	ST	66.1 ± 7.6	8.8 ± 0.4	0.70 ± 0.08	60.5 ± 4.6
	30P1	33.5 ± 2.1	10.8 ± 0.7	1.20 ± 0.02	33.3 ± 1.1
	50P1	35.0 ± 1.9	10.9 ± 0.7	1.20 ± 0.02	33.0 ± 1.6
	150P1	32.2 ± 2.7	11.6 ± 0.3	1.41 ± 0.04	22.1 ± 2.4
	150P3	32.4 ± 2.1	12.0 ± 0.2	1.45 ± 0.13	20.4 ± 6.9

**Figure 6:**
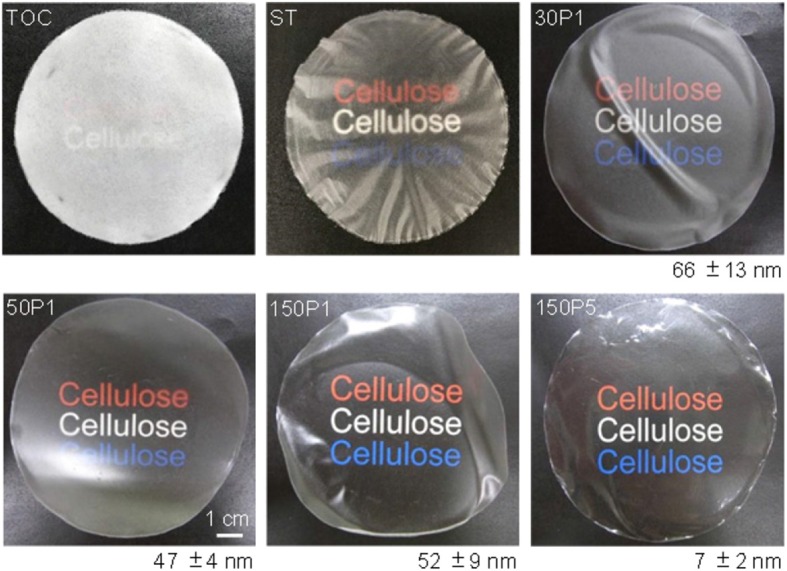
Photographs of the TOC sheet and films prepared from fibrillated TOC/water dispersions and the average surface roughness values with standard deviations.

Photographs of the sheet and films are shown in Figure 6 along with average surface roughness values determined from AFM images. The TOC sheet was opaque, like a paper sheet consisting of wood fibers. The ST film consisted of uneven and opaque fiber-rich and translucent regions. The film prepared from the TOC/water dispersion with the highest degree of fibrillation had the highest transparency and surface smoothness.

**Figure 7:**
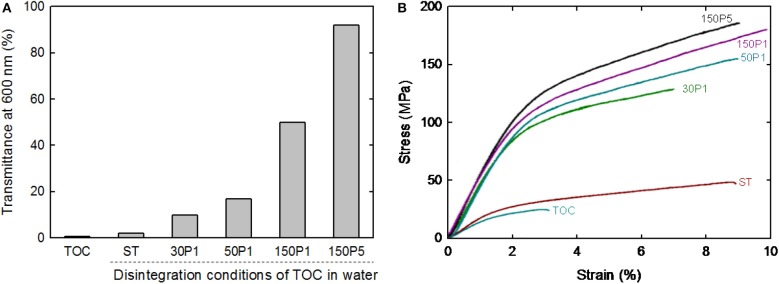
**(A)** Light transmittance at 600 nm and **(B)** strain–stress curves of the TOC sheet and films prepared from fibrillated TOC/water dispersions.

The light transmittance values of the sheet and films at 600 nm wavelength normalized to 50 μm thickness are shown in Figure 7A. The film transparency increased with increasing degree of fibrillation of the TOC/water slurry. The strain–stress curves of the sheet and films are shown in Figure 7B. The tensile strength and Young's modulus clearly increased with increasing degree of fibrillation of the TOC/water slurry. The mechanical properties, including the tensile strength, Young's modulus, elongation at break, and work of fracture, are given in Table 2. These four mechanical properties explicitly improved with increasing degree of fibrillation of the TOC/water slurry. The tensile strength and Young's modulus of the films showed almost linear relationships with the film density. Therefore, the various disintegration conditions of the TOC/water slurry lead to different filtration times and film densities, resulting in the films having diverse mechanical properties.

**Table 2:** Mechanical properties of the TOC sheet and films prepared from fibrillated TOC/water dispersions.

	**Disintegration condition**	**Tensile strength** **(MPa)**	**Young's modulus** **(GPa)**	**Elongation at break (%)**	**Work of fracture** **(MJ/m^**3**^)**
TOC sheet	–	20.1 ± 2.3	1.6 ± 0.2	2.9 ± 0.6	0.4 ± 0.1
Films prepared from fibrillated TOC	ST	42.5 ± 10.5	1.9 ± 0.8	8.6 ± 1.4	2.6 ± 0.6
	30P1	115.2 ± 8.8	5.7 ± 0.5	6.7 ± 0.8	5.5 ± 1.0
	50P1	139.0 ± 7.0	6.5 ± 0.8	8.3 ± 0.9	8.1 ± 1.1
	150P1	167.7 ± 9.8	7.1 ± 0.6	8.7 ± 0.9	10.2 ± 1.5
	150P3	181.0 ± 15.0	7.3 ± 0.9	9.3 ± 1.1	11.7 ± 1.6

**Figure 8:**
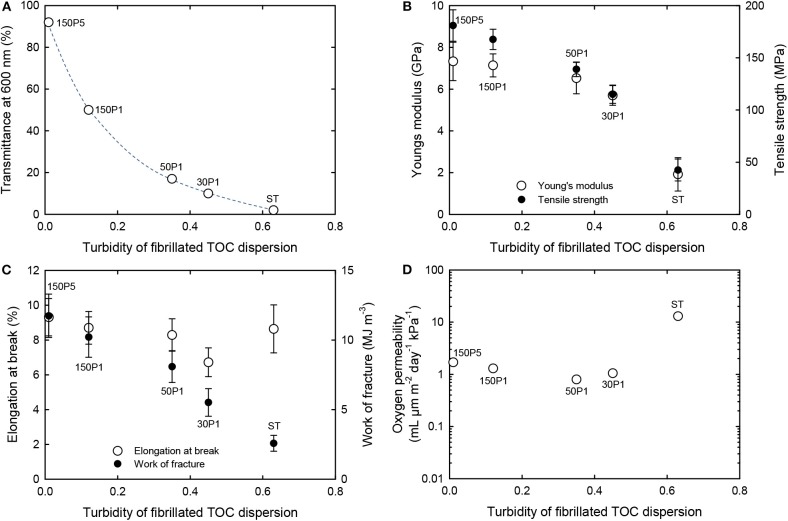
Relationships between turbidity of the TOC/water dispersion and the **(A)** light transmittance, **(B)** Young's modulus and tensile strength, **(C)** elongation at break and work of fracture, and **(D)** oxygen permeability of films prepared from fibrillated TOC/water dispersions.

The optical, mechanical, and oxygen permeability properties of the films are plotted as a function of the turbidity of the dispersion in Figure 8. The optical transparency, tensile strength, Young's modulus, and work of fracture of the films increased with decreasing turbidity of the fibrillated TOC/water dispersion or increasing degree of fibrillation. However, the elongation at break exhibited no clear relation with the dispersion turbidity. The oxygen permeability values of the 150P5, 150P1, 50P1, and 30P1 films were as low as 1–2 ml μm m^−2^ day^−1^ kPa^−1^ at 23°C and 50% RH, whereas that of the ST film was almost one order higher than those of the other films. This indicates that the good oxygen-barrier properties of the films were caused by the nanofibrillated TOCN components partly formed from the TOC fibers during the high-pressure homogenization process and present in the films together with large fibrils and fibril bundles.

## Conclusions

The time required to prepare TEMPO-oxidized nanocellulose films by membrane filtration can be controlled by controlling the degree of fibrillation of the TOC/water slurry. The filtration time to prepare films from the dispersions by membrane filtration can be shortened by selecting the dispersions with lower degree of fibrillation. Because low degrees of fibrillation of the TOC/water slurry cause only partial nanofibrillation of TOC to form CNNeW structures, the as-prepared dispersions had high turbidity values and contained significant amounts of unfibrillated fractions. When the degree of fibrillation of the TOC/water slurry was controlled from zero (i.e., the original TOC fibers) to 100% nanofibrillation to prepare a transparent TOCN dispersion, the filtration time increased from 0 min to 2.6 h with 5% yield loss. The film density and porosity significantly differed depending on the degree of fibrillation of the TOC/water slurry, and the optical and mechanical properties of the films remarkably differed. However, the oxygen-barrier properties of the films were relatively low and similar among the films prepared from the TOC/water slurry by high-pressure homogenization under various conditions. The films prepared from fibrillated TOC/water dispersions with different degrees of fibrillation can be used to prepare CNNeW films with high carboxylate contents, high porosities, and sufficiently low oxygen permeabilities considering the efficient water removal processes from the water-rich dispersions.

## Data Availability Statement

The raw data supporting the conclusions of this article will be made available by the authors, without undue reservation, to any qualified researcher.

## Author Contributions

AI designed the experiments and wrote the manuscript. MW carried out experiments. All authors analyzed experimental results, and approved the submitted version.

### Conflict of Interest

The authors declare that the research was conducted in the absence of any commercial or financial relationships that could be construed as a potential conflict of interest.

## References

[B1] Abe K. Iwamoto S. Yano H. (2007). Obtaining cellulose nanofibers with a uniform width of 15 nm from wood. Biomacromolecules 8, 3276–3278. 10.1021/bm700624p 17784769

[B2] Daicho K. Saito T. Fujisawa S. Isogai A. (2018). The crystallinity of nanocellulose: dispersion-induced disordering of the grain boundary in biologically structured cellulose. ACS Appl. Nano Mater. 1, 5774–5785. 10.1021/acsanm.8b01438

[B3] de Nooy A. E. J. Besemer A. C. van Bekkum H. (1995). Highly selective nitroxyl radical-mediated oxidation of primary alcohol groups in water-soluble glucans. Carbohydr. Res. 269, 89–98. 10.1016/0008-6215(94)00343-E 11072842

[B4] de Nooy A. E. J. Besemer A. C. van Bekkum H. van Dijk J. A. P. P. Smit J. A. M. (1996). TEMPO-mediated oxidation of pullulan and influence of ionic strength and linear charge density on the dimensions of the obtained polyelectrolyte chains. Macromolecules 29:65416547 10.1021/ma960492t

[B5] De Souza Lima M. M. Borsali R. (2004). Rodlike cellulose microcrystals: structure, properties, and applications. Macromol. Rapid Commun. 25, 771–787. 10.1002/marc.200300268

[B6] Eichhorn S. J. Dufuresne A. Aranguren M. Marcovich N. E. Capadona J. R. Rowan S. J. (2010). Review: current international research into cellulose nanofibres and nanocomposites. J. Mater. Sci. 45, 1–33. 10.1007/s10853-009-3874-0 PMC7307936 32836382

[B7] Fraschini C. Chauve G. Bouchard J. (2017). TEMPO-mediated surface oxidation of cellulose nanocrystals (CNCs). Cellulose 24:2775 10.1007/s10570-017-1319-5

[B8] Fukuzumi H. Fujisawa S. Saito T. Isogai A. (2013). Selective permeation of hydrogen gas using cellulose nanofibril film. Biomacromolecules 14, 1705–1709. 10.1021/bm400377e 23594396

[B9] Fukuzumi H. Saito T. Iwata T. Kumamoto Y. Isogai A. (2009). Transparent and high gas barrier films of cellulose nanofibers prepared by TEMPO-mediated oxidation. Biomacromolecules 10, 162–165. 10.1021/bm801065u 19055320

[B10] Henriksson M. Berglund L. A. Isaksson P. Lindström T. Nishino T. (2008). Cellulose nanopaper structures of high toughness. Biomacromolecules 9, 1579–1585. 10.1021/bm800038n 18498189

[B11] Henriksson M. Henriksson G. Berglund L. A. Lindström T. (2007). An environmentally friendly method for enzyme-assisted preparation of microfibrillated cellulose (MFC) nanofibers. Eur. Polym. J. 43, 3434–3441. 10.1016/j.eurpolymj.2007.05.038

[B12] Hirota M. Furihata K. Saito T. Kawada T. Isogai A. (2010). Glucose/glucuronic acid alternating copolysaccharide prepared from TEMPO-oxidized native celluloses by surface-peeling. Angew. Chem. Int. Ed. 49, 7670–7672. 10.1002/anie.201003848 20839205

[B13] Isogai A. Hänninen T. Fujisawa S. Saito T. (2018). Review: catalytic oxidation of cellulose with nitroxyl radicals under aqueous conditions. Prog. Polym. Sci. 86, 122–148. 10.1016/j.progpolymsci.2018.07.007

[B14] Isogai A. Saito T. Fukuzumi H (2011). TEMPO-oxidized cellulose nanofibers. Nanoscale 3, 71–85. 10.1039/c0nr00583e 20957280

[B15] Isogai A. Zhou Y. (2019). Diverse nanocelluloses prepared from TEMPO-oxidized wood cellulose fibers: nanonetworks, nanofibers, and nanocrystals. Curr. Opin. Solid State Mater. Sci. 23, 101–106. 10.1016/j.cossms.2019.01.001

[B16] Klemm D. Kramer F. Moritz S. Lindström T. Ankerfors M. Gray D.. (2011). Nanocelluloses: a new family of nature-based materials. Angew. Chem. Int. Ed. 50, 5438–5466. 10.1002/anie.201001273 21598362

[B17] Liu A. Walther A. Ikkala O. Belova L. Berglund L. A. (2011). Clay nanopaper with tough cellulose nanofiber matrix for fire retardancy and gas barrier functions. Biomacromolecules 12, 633–641. 10.1021/bm101296z 21291221

[B18] Moon R. J. Martini A. Nairn J. Simonsen J. Youngblood J. (2011). Cellulose nanomaterials review: structure, properties and nanocomposites. Chem. Soc. Rev. 40, 3941–3994. 10.1039/c0cs00108b 21566801

[B19] Nakagaito A. N. Yano H. (2005). Novel high-strength biocomposites based on microfibrillated cellulose having nano-order-unit web-like network structure. Appl. Phys. A 80, 155–159. 10.1007/s00339-003-2225-2

[B20] Nakagaito A. N. Yano H. (2008). The effect of fiber content on the mechanical and thermal expansion properties of biocomposites based on microfibrillated cellulose. Cellulose 15, 555–559. 10.1007/s10570-008-9212-x

[B21] Nemoto J. Saito T. Isogai A. (2015). Simple freeze-drying procedure for producing nanocellulose aerogel-containing, high-performance air filters. ACS Appl. Mater. Interfaces 7, 198909–198915. 10.1021/acsami.5b05841 26301859

[B22] Okita Y. Saito T. Isogai A. (2009). TEMPO-mediated oxidation of softwood thermomechanical pulp. Holzforschung 63, 529–535. 10.1515/HF.2009.096

[B23] Pääkkö M. Ankerfors M. Kosonen H. Nykänen A. Ahola S. Osterberg M.. (2007). Enzymatic hydrolysis combined with mechanical shearing and high-pressure homogenization for nanoscale cellulose fibrils and strong gels. Biomacromolecules 8, 1934–1941. 10.1021/bm061215p 17474776

[B24] Saito T. Isogai A. (2004). TEMPO-mediated oxidation of native cellulose. The effect of oxidation conditions on chemical and crystal Structures of the water-insoluble fractions. Biomacromolecules 5, 1983–3989. 10.1021/bm0497769 15360314

[B25] Sakai K. Kobayashi Y. Saito T. Isogai A. (2016). Partitioned airs at microscale and nanoscale: thermal diffusivity in ultrahigh porosity solids of nanocellulose. Sci. Rep. 6:20434. 10.1038/srep20434 26830144 PMC4735846

[B26] Sehaqui H. Ezekiel Mushi N. Morimune S. Salajkova M. Nishino T. Berglund L. A. (2012). Cellulose nanofiber orientation in nanopaper and nanocomposites by cold drawing. ACS Appl. Mater. Interfaces 4, 1043–1149. 10.1021/am2016766 22257144

[B27] Sehaqui H. Liu A. Zhou Q. Berglund L. A. (2010). Fast preparation procedure for large, flat cellulose and cellulose/inorganic nanopaper structures. Biomacromolecules 11, 2195–2198. 10.1021/bm100490s 20698565

[B28] Sehaqui H. Zhou Q. Ikkala O. Berglund L. A. (2011). Strong and tough cellulose nanopaper with high specific surface area and porosity. Biomacromolecules 12, 3638–3644. 10.1021/bm2008907 21888417

[B29] Shimizu M. Saito T. Isogai A. (2016). Water-resistant and high oxygen-barrier nanocellulose films with interfibrillar cross-linkages formed through multivalent metal ions. J. Membr. Sci. 500, 1–7. 10.1016/j.memsci.2015.11.002

[B30] Svagan A. J. Samir M. A. Berglund L. A. (2007). Biomimetic polysaccharide nanocomposites of high cellulose content and high toughness. Biomacromolecules 8, 2556–2563. 10.1021/bm0703160 17655354

[B31] Varanashi S. Batchelor W. J. (2013). Rapid preparation of cellulose nanofiber sheet. Cellulose 20, 211–215. 10.1007/s10570-012-9794-1

[B32] Wågberg L. Decher G. Norgren M. Lindström T. Ankerfors M. Axnäs K. (2008). The build-up of polyelectrolyte multilayers of microfibrillated cellulose and cationic polyelectrolytes. Langmuir 24, 784–795. 10.1021/la702481v 18186655

[B33] Wu C. N. Yang Q. Takeuchi M. Saito T. Isogai A. (2014). Highly tough and transparent layered composites of nanocellulose and synthetic silicate. Nanoscale 6, 392–399. 10.1039/c3nr04102f 24201761

[B34] Yang Q. Saito T. Berglund L. A. Isogai A. (2015). Cellulose nanofibrils improve the properties of all-cellulose composites by the nano-reinforcement mechanism and nanofibril-induced crystallization. Nanoscale 7, 17957–17963. 10.1039/c5nr05511c 26465589

[B35] Zhao M. Ansari F. Takeuchi M. Shimizu M. Saito T. Berglund L. A. (2018). Nematic structuring of transparent and multifunctional nanocellulose papers. Nanoscale Horizn. 3, 28–34. 10.1039/C7NH00104E 32254107

[B36] Zhou Y. Fujisawa S. Saito T. Isogai A. (2019). Characterization of concentration-dependent gelation Behavior of aqueous 2,2,6,6-tetramethylpiperidine-1-oxyl–cellulose nanocrystal dispersions using dynamic light scattering. Biomacromolecules 20, 750–757. 10.1021/acs.biomac.8b01689 30557007

[B37] Zhou Y. Saito T. Bergström L. Isogai A. (2018). Acid-free preparation of cellulose nanocrystals by TEMPO oxidation and subsequent cavitation. Biomacromolecules 19, 633–639. 10.1021/acs.biomac.7b01730 29283555

